# Safety and efficacy of ferric citrate in phosphate reduction and iron supplementation in patients with chronic kidney disease

**DOI:** 10.18632/oncotarget.21990

**Published:** 2017-10-20

**Authors:** Mei-Yi Wu, Ying-Chun Chen, Chun-Hung Lin, Yun-Chun Wu, Yu-Kang Tu, Der-Cherng Tarng

**Affiliations:** ^1^ Division of Nephrology, Department of Internal Medicine, Shuang Ho Hospital, Taipei Medical University, Taipei, Taiwan; ^2^ Department of Internal Medicine, School of Medicine, College of Medicine, Taipei Medical University, Taipei, Taiwan; ^3^ Graduate Institute of Epidemiology and Preventive Medicine, College of Public Health, National Taiwan University, Taipei, Taiwan; ^4^ Department of Pharmacy, Taipei Medical University, Shuang Ho Hospital, Taipei, Taiwan; ^5^ Department of Education, School of Medicine, College of Medicine, Taipei Medical University, Taipei, Taiwan; ^6^ Institute of Clinical Medicine, National Yang-Ming University, Taipei, Taiwan; ^7^ Department and Institute of Physiology, National Yang-Ming University, Taipei, Taiwan; ^8^ Division of Nephrology, Department of Medicine, Taipei Veterans General Hospital, Taipei, Taiwan

**Keywords:** ferric citrate, chronic kidney disease, phosphate binder, anemia, meta-analysis

## Abstract

Ferric citrate has been reported to have the potential to reduce phosphate and increase iron availability in patients with chronic kidney disease. In the present study, we evaluated its safety and efficacy in phosphate reduction and iron supplementation in chronic kidney disease stage 3-5 requiring dialysis patients. We systematically searched for clinical trials published in PubMed, Medline, and Cochrane databases. Only randomized controlled trials on the effects of ferric citrate in chronic kidney disease stage 3–5 requiring dialysis patients were selected. The primary outcomes were changes in serum phosphate, calcium, and anemia-related parameters. The secondary outcomes were the adverse effects of ferric citrate. Nine studies providing data on 1755 patients were included in the meta-analysis. Ferric citrate significantly reduced serum phosphate compared with placebo (mean difference, –1.39; 95% confidence interval, –2.12 to –0.66) and had a non-inferior effect compared with active treatment. Furthermore, ferric citrate significantly improved hemoglobin, transferrin saturation and ferritin. Adverse effects of constipation did not differ significantly between ferric citrate and placebo or active treatment. This review provides evidence that ferric citrate effectively alleviates hyperphosphatemia and iron deficiency in patients with chronic kidney disease stage 3–5 requiring dialysis patients. However, the included studies did not have cardiovascular complications or mortality information and could not assess whether ferric citrate affected the risk of all-cause death or cardiovascular complications in patients with chronic kidney disease. Further studies are required to assess whether the long-term use of ferric citrate can reduce the risk of cardiovascular events and all-cause mortality.

## INTRODUCTION

Chronic kidney disease (CKD), a worldwide health burden, consumes disproportionate health care resources. Anemia, secondary hyperparathyroidism, and cardiovascular disease are highly prevalent in patients with CKD. Anemia and hyperphosphatemia are potentially modifiable risk factors for CKD progression and mortality [1, 2]. Furthermore, anemia is frequently associated with a higher risk of cardiovascular events and all-cause mortality [3, 4]. The causes of anemia in patients with CKD include erythropoietin and iron deficiency, inflammation-malnutrition complex, shortened red blood cell lifespan, and increased blood loss [5]. Treatment with erythropoiesis-stimulating agents (ESAs) benefited patients with CKD and reduced the need for frequent blood transfusion. Hyperphosphatemia causes vascular and valvular calcification, thereby increasing the risk of peripheral arterial disease and hospitalization for heart failure in patients with CKD.

The advent of ESAs has effectively alleviated anemia in patients with CKD, and the combination of iron and ESAs can reduce the dose of ESA. The 2012 Kidney Disease: Improving Global Outcomes guidelines recommended iron therapy for anemia management when ferritin < 500 ng/mL and transferrin saturation (TSAT) < 30% [6]. However, concerns have been raised regarding the association of cardiovascular complications and infections with long-term intravenous, high-dose iron supplementation. Moreover, it is convenient to administer oral iron to patients with non-dialysis CKD and those receiving peritoneal dialysis [7].

The current recommendation for hyperphosphatemia management in patients with CKD stage 3–5 requiring dialysis (3–5D) involves reducing elevated phosphate to the normal range and avoiding hypercalcemia. General principles for reducing hyperphosphatemia were dietary phosphate restriction and phosphate binder use. There are many classes of phosphate binders, including calcium-based phosphate binders and calcium-free binders (sevelamer, lanthanum, and iron-based phosphate binders) A restricted dose of calcium-based phosphate binders is recommended for patients with CKD stage 3-5D comorbid with hypercalcemia [8]. Calcium-free binders may halt the progression of vascular calcifications compared with calcium-based agents [9].

Ferric citrate (FC), an iron-based intestinal phosphate binder, was reported to reduce serum phosphate and increase hemoglobin (Hb) levels by repleting iron stores in patients with CKD patients [10]. These findings were only observed in a few small- and medium-sized prospective randomized controlled trials (RCTs), in which FC was used for achieving reduction in serum phosphate and improvement in iron stores and anemia in patients with CKD stage 3–5D. Guidelines recommend iron supplements, either in the oral form or intravenous formulation, for patients with CKD having an absolute or functional iron deficiency [11, 12]. Oral iron supplement is less expensive, is easier to be administered, and is typically recommended as the first-line therapy for patients with non-dialysis CKD having iron deficiency. As an iron-based phosphate binder, FC may improve iron-related parameters, subsequently improving anemia in patients with CKD. Therefore, we conducted a systematic review and meta-analysis of all available RCTs to determine the safety and efficacy of FC for reducing phosphate and alleviating anemia and iron parameters in patients with CKD stage 3–5D.

## RESULTS

### Trial flow and study characteristics

**Figure 1:**
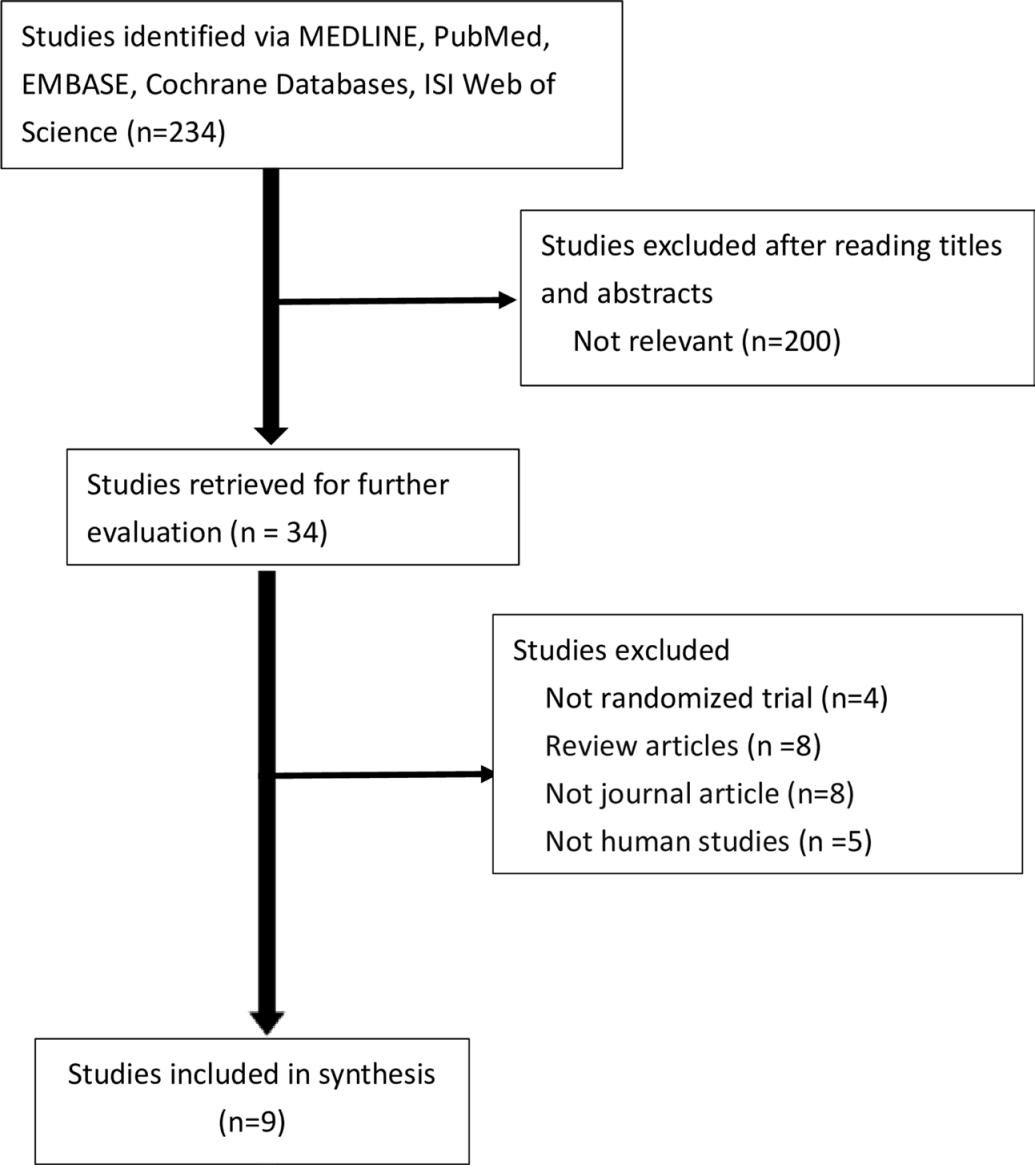
Flowchart for selection of trials

We screened 234 studies retrieved from our literature search (Figure 1). Finally, 225 studies were excluded; thus, 9 RCTs were analyzed [13–21]. A total of 1755 patients were included from the included RCTs. The effects of FC were assessed compared with those of placebo in 6 trials and with those of the active treatment of sevelamer in 3 trials and calcium carbonate in 1 trial (Table 1) [15–18]. The study duration in all trials ranged from 4 to 52 weeks. The study enrolled patients with CKD stage 3–5 or those who received dialysis (hemodialysis or peritoneal dialysis). The trial characteristics are summarized in Table 1. Baseline characteristics of patients in all the included studies are summarized in Table 1. The studies were published between 2002 and 2015, and had sample sizes ranging from 45 to 441. The daily dosage of FC ranged from 1.5–6.0 g/day. Patients with CKD stage 3–5D were evaluated in all trials. The modality of dialysis was hemodialysis or peritoneal dialysis. Mean age of patients in these trials ranged from 52.5 to 66 years. Baseline characteristics and phosphate were similar between the two treatment groups. But serum phosphate at baseline was quite different across trials, ranging from 4.23 to 7.93 mg/dl. The dosages of ESA and intravenous iron were adjusted for according to various protocols. We used a random effects model for assessing clinical heterogeneity.

**Table 1:** Characteristics of included randomized controlled trials

Study	Country	Intervention Drug (dose/d)	Comparator Drug(s) (dose/d)	Treatment duration (wk)	Patients n (% of male)	BL CKD stage	Age (y)	Serum phosphate (mg/dl)	Inclusion criteria (Iron parameters)
**Block [2015]**	US	FC 3 g (mean, 5.1 g)	Placebo (mean, 5.2 g)	12	I: 75 (31) C: 74 (38)	3–5	I: 66 ± 12 C: 64 ± 14	I: 4.5 ± 0.6 C: 4.7 ± 0.6	Ferritin ≤ 300 TSAT ≤ 30%
**Fishbane [2017]**	US	FC 3–6 g (mean, 5.1 g)	Placebo (mean, 5.2 g)	16	I: 117 (35.0) C: 116 (38.8)	3–5	I: 65.6 ± 11.2 C: 65.2 ± 13.1	I: 4.23 ± 0.91 C: 4.12 ± 0.68	Ferritin ≤ 200 TSAT ≤ 25%
**Lee [2015]**	TW	I1: FC 4 g I2: FC 6 g	Placebo	8	I1: 75 (37.3) I2: 72 (43.1) C: 36 (30.6)	5D	I1: 53.4 ± 11.7 I2: 56.4 ± 10.5 C: 53.0 ± 11.8	I1: 6.96 ± 1.08 I2: 6.95 ± 1.15 C: 7.37 ± 1.26	Ferritin ≤ 800
**Van Buren [2015]**	US	FC (median, 8 g)	C1: Calcium acetate C2: Sevelamer carbonate C3: C1 and C2	52	I: 292 (62.7) C: 149 (58.4)	5D*	I: 54.9 ± 13.4 C: 53.7 ± 13.0	I: 7.2 [6.3–8.3] C: 7.4 [6.2-5.8]	Ferritin < 1000 TSAT < 50%
**Lewis [2015]**	US	FC (median, 8 g)	Placebo	4	I: 96 (72.9) C: 96 (49.0)	5D*	I: 54.0 [45.0–62.5] C: 56.0 [48.5–62.0]	I: 5.1 [4.3–6.0] C: 5.3 [6.2–8.5]	Ferritin < 1000 TSAT < 50%
**Yang [2002]**	TW	FC 3 g	Calcium carbonate 3 g	4	45 (48.9)	5D	52.5 ± 11.8	I: 6.7 ± 1.9 C: 7.2 ± 1.9	Ferritin normal range TSAT normal range
**Yokoyama [2012]**	JP	I1: FC 1.5 g I2: FC 3 g I3: FC 6 g	Placebo	4	I1: 49 (61.2) I2: 50 (64.0) I3: 45 (68.9) C: 48 (56.3)	5D	I1: 60.9 ± 8.9 I2: 58.6 ± 12.3 I3: 58.1 ±10.6 C: 62.7 ± 11.0	I1: 7.69 ± 1.28 I2: 7.84 ± 1.29 I3: 7.93 ±1.40 C: 7.84 ± 1.20	Ferritin ≤ 300
**Yokoyama [2014–1]**	JP	FC 1.5–6 g	Placebo	12	I: 60 (57.9) C: 30 (58.6)	3–5	I: 65.3 ± 10.2 C: 64.6 ± 13.5	I: 5.66 ± 0.75 C: 5.57 ± 0.63	Ferritin ≤ 500 TSAT ≤ 50%
**Yokoyama [2014–2]**	JP	FC 1.5–6 g	Sevelamer hydrochloride 3–9 g	12	I: 116 (63.5) C: 114 (65.5)	5D	I: 60.2 ± 10.7 C: 61.4 ± 9.5	I: 7.84 ± 1.36 C: 7.80 ± 1.32	Ferritin ≤ 500 TSAT ≤ 50%

Abbreviations: 5D, chronic kidney disease treated with dialysis; BL, baseline; FC, ferric citrate; JP, Japan; TSAT, transferrin saturation; TW, Taiwan; US, United States.

Notes: Values for continuous variables are given as mean ± standard deviation or median [interquartile range].

^*^Included hemodialysis or peritoneal dialysis.

### Study quality

Among the 9 RCTs, 4 clearly documented the randomization process and allocation concealment [13, 17, 18, 21], whereas an imbalance in serum phosphate was reported in a crossover study, raising some concerns on the randomization process [16]. An open-label design was used in 50% of the enrolled studies [15–18], which can introduce investigator or subject bias associated with the reporting of adverse effects. However, the primary outcomes were all laboratory values and unlikely to be influenced by a lack of blinding. Data in all included studies, except the one by Lee et al. [19], were analyzed based on the intention-to-treat or modified intention-to-treat principle. Table 2 reports the risk-of-bias assessment.

**Table 2:** Assessment of the methodological quality of included studies

Study	Allocation generation	Allocation concealment	Blinding	Incomplete outcome data	Selective reporting	Other bias
**Block [2015]**	Low risk	Low risk	Double blind	mITT	Low risk	
**Fishbane [2017]**	Unclear	Unclear	Double blind	mITT	Low risk	
**Lee [2015]**	Unclear	Unclear	Double blind	PP, Dropout rate: Placebo (66.7%) v.s. FC (18.4%)	Low risk	
**Lewis [2015]**	Low risk	Low risk	Open-label	ITT	Low risk	
**Van Buren [2015]**	Low risk	Low risk	Open-label	ITT	Low risk	
**Yang [2002]**	High risk	Unclear	Open-label	Unclear	Low risk	Imbalance serum phosphate level after wash-out period
**Yokoyama [2012]**	Low risk	Low risk	Double blind	ITT	Low risk	
**Yokoyama [2014–1]**	Unclear	Unclear	Double blind	ITT	Low risk	
**Yokoyama [2014–2]**	Unclear	Unclear	Open-label	ITT	Low risk	

Abbreviations: FC, ferric citrate; ITT, intention to treat; mITT, modified intention-to-treat; PP, per-protocol.

### Publication bias

Publication bias was not assessed because the number of included trials was inadequate to appropriately produce a funnel plot.

### Changes in serum phosphate, calcium, intact-parathyroid hormone, and fibroblast growth factor 23

**Figure 2:**
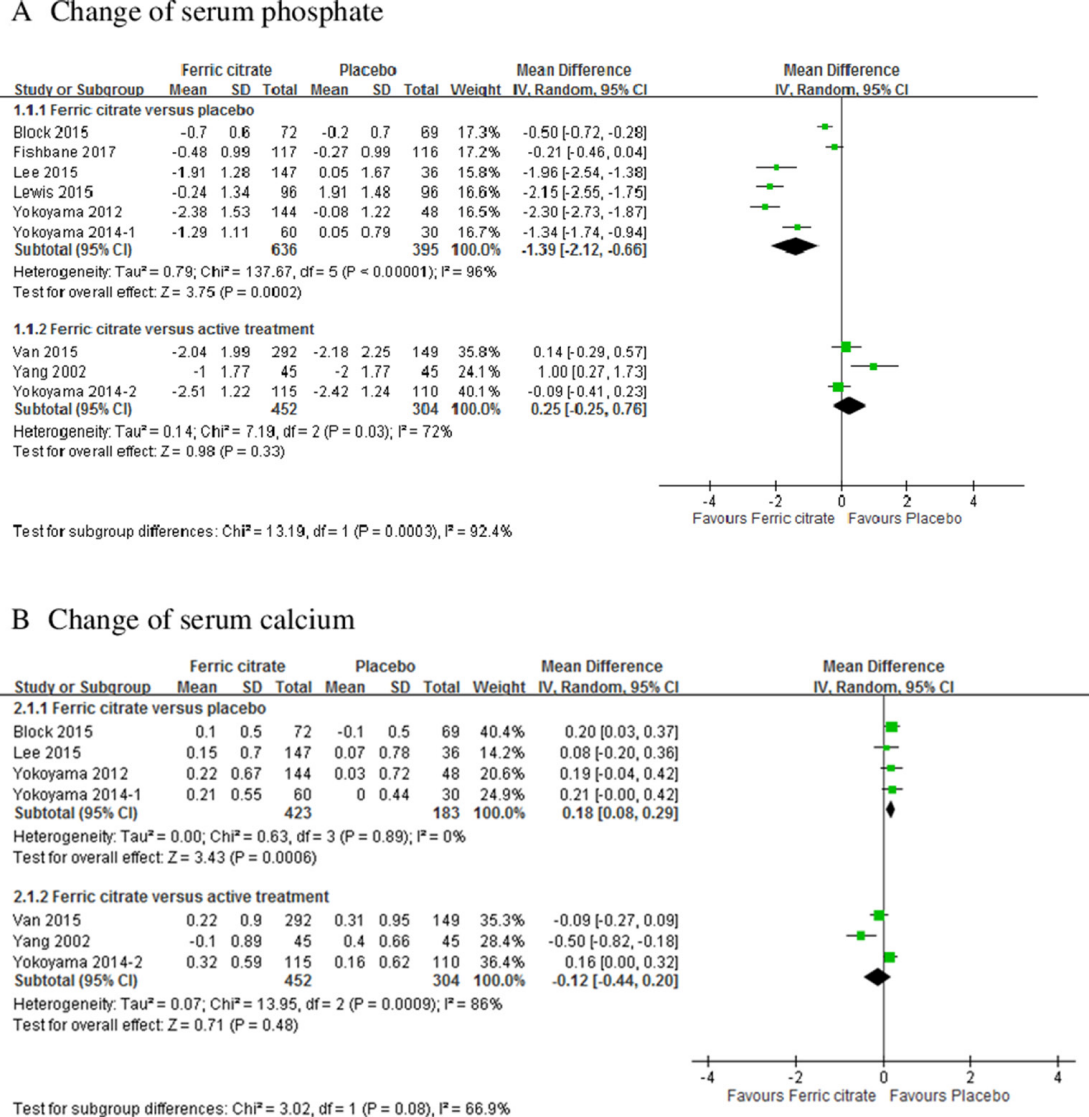

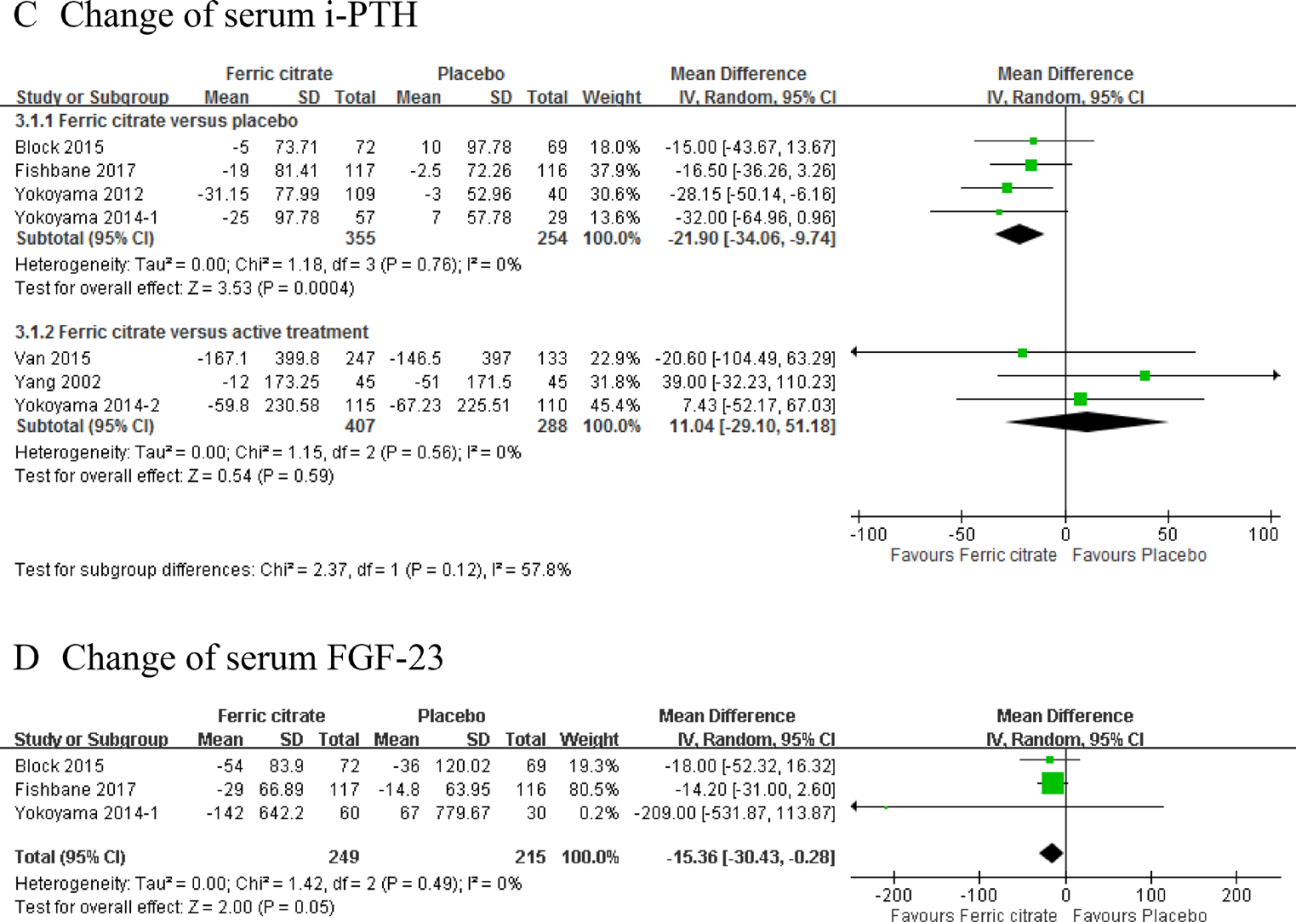
Forest plot of comparisons in ferric citrate versus placebo or ferric citrate versus active treatment Outcomes included (**A**) change of serum phosphate; (**B**) change of serum calcium; (**C**) change of serum intact parathyroid hormone (i-PTH); and (**D**) change of serum fibroblast growth factor (FGF)-23.

In a meta-analysis of 6 studies, the weighted mean change in serum phosphate from the baseline to the last measurement was: 1.39 (95% confidence interval [CI], –2.12 to –0.66) in patients treated with FC versus those receiving placebo [13, 14, 18–21]. No statistical significance was observed in serum phosphate change between the FC and active treatment groups (mean difference [MD]: 0.25, 95% CI, –0.25 to 0.76) (Figure 2A). Seven trials including 1362 patients reported the change in serum calcium [14–18, 20, 22]. The weighted mean change in serum calcium from the baseline to the last measurement was 0.18 (95% CI, 0.08– 0.29) in patients treated with FC compared with those receiving placebo. No statistical significance was observed in calcium change between the FC and active treatment groups (MD: –0.12, 95% CI, –0.44 to 0.2; Figure 2B). Seven trials including 1304 patients reported changes in serum intact-parathyroid hormone (i-PTH) [13–17, 20, 21]. The pre-specified subgroup analysis according to the comparison group revealed that the weighted mean change in serum i-PTH from the baseline to the last measurement was –21.9 (95% CI, –34.06 to –9.74) in patients treated with FC compared with those receiving placebo. No statistical significance was observed in i-PTH changes between the FC and active treatment groups (MD: 11.04, 95% CI, –29.1 to 51.18; Figure 2C). The effects of FC in reducing fibroblast growth factor (FGF)–23 was significant compared with the placebo group (MD: –15.36, 95% CI, –30.43 to –0.28; Figure 2D) [14, 20, 21].

### Changes in Hb, TSAT, and ferritin

**Figure 3:**
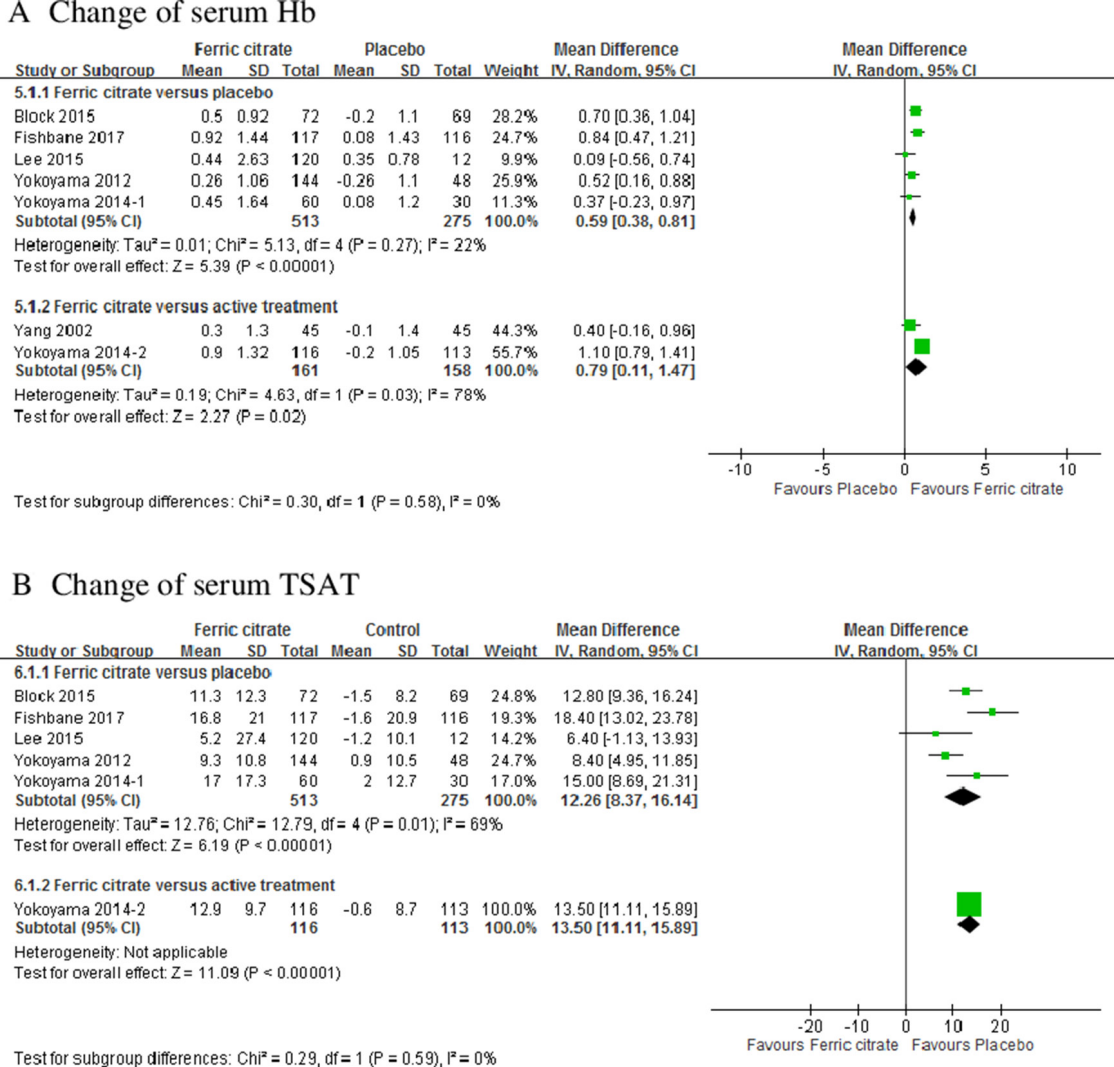

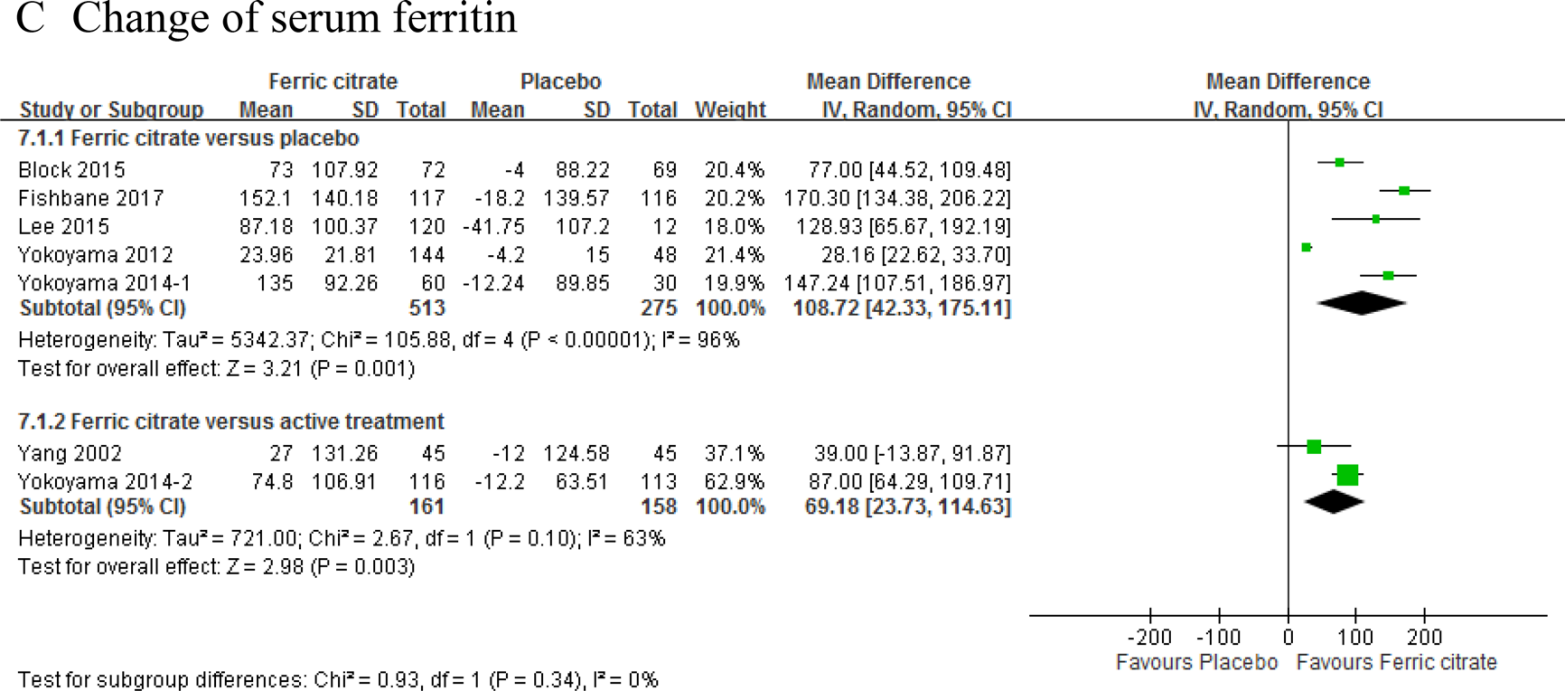
Forest plot of comparisons in ferric citrate versus placebo or ferric citrate versus active treatment Outcomes included (A) change of hemoglobin; (B) change of transferrin saturation; and (C) change of serum ferritin.

Changes in the Hb level from the baseline to the end of the study were higher in patients treated with FC (MD: 0.59; 95% CI, 0.38 to 0.81, 5 trials) than in those receiving placebo [13–16, 19–21]. The effects of FC in increasing Hb levels were similar to those of different active treatments (MD: 0.79; 95% CI, 0.11 –1.47, 2 trials; Figure 3A). TSAT levels were significantly higher in the FC group than in the placebo group (MD: 12.26; 95% CI, 8.37 –16.14; 5 trials) and active treatment groups (MD: 13.5; 95% CI, 11.11 –15.89, 1 trial; Figure 3B). Ferritin levels were significantly higher in the FC group than in the placebo group (MD: 108.72; 95% CI, 42.33 –175.11; 5 trials; Figure 3C) [13, 14, 16, 19–21]. No statistical significance was observed in changes in ferritin levels between the FC and active treatment groups.

### Risk of adverse events

**Figure 4:**
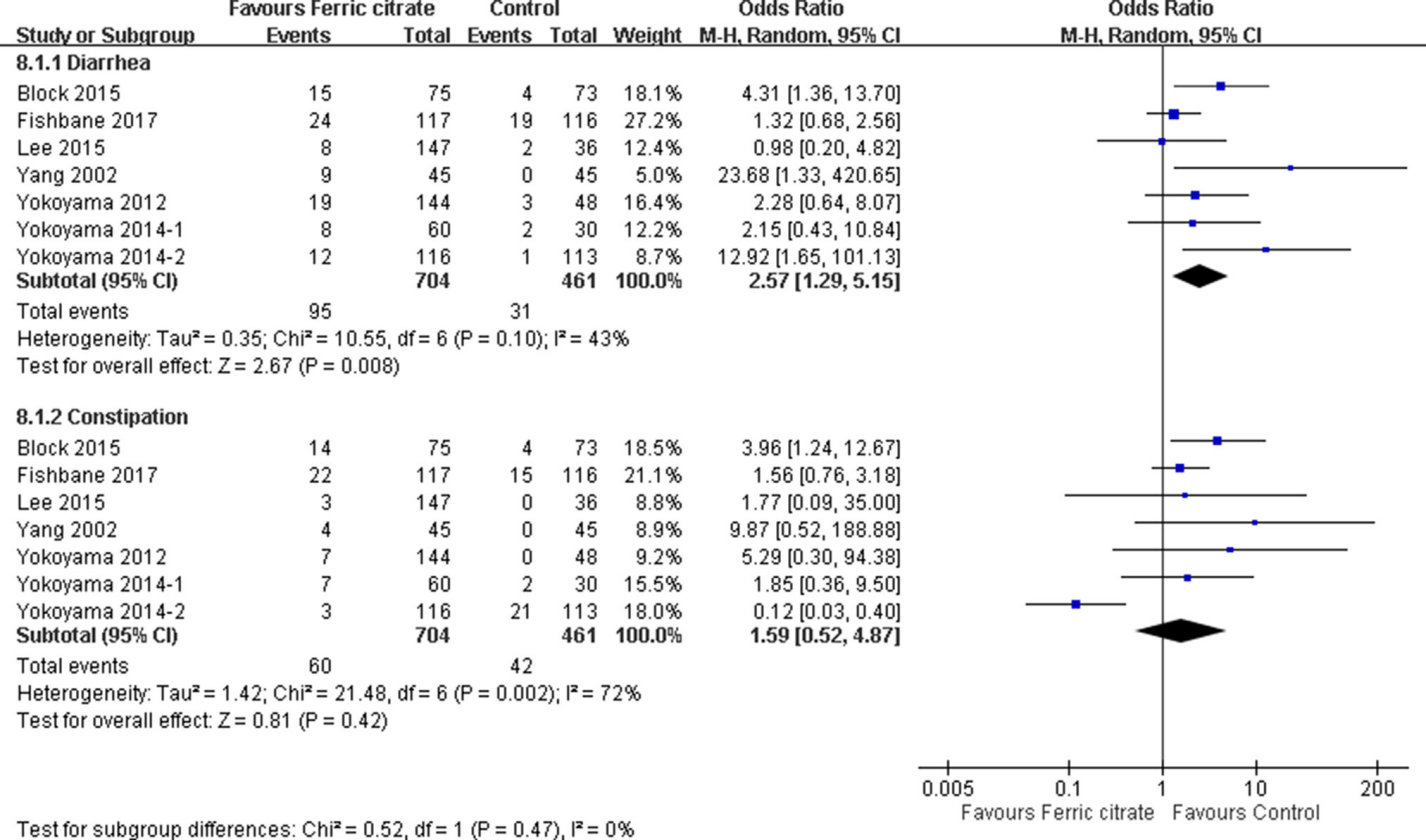
Forest plot of comparison in ferric citrate versus control Outcomes included adverse effects of diarrhea and constipation.

The incidence of diarrhea and constipation was analyzed among 1165 patients in 7 RCTs (Figure 4). Among patients treated with FC, the summary OR of diarrhea was 2.57 (95% CI 1.29–5.15) using a random effects model (heterogeneity I^2^ = 43%, *p* = 0.10) [14–17, 20–22]. Diarrhea occurred in 13.5% (95 of 704) participants receiving FC. Furthermore, in patients treated with FC, the summary OR of constipation was 1.59 (95% CI, 0.52–4.87) using a random effects model (heterogeneity I^2^ = 72%, *p* = 0.002). Constipation occurred in 8.5% (60 of 704) participants receiving FC.

## DISCUSSION

The present systematic review of 9 RCTs involving 1755 participants provides evidence suggesting that a course of FC treatment in patients with CKD stage 3–5D is beneficial in alleviating hyperphosphatemia and anemia. Consistent with this finding, FC reduced hyperphosphatemia 1.39 mg/dL (95% CI, –2.12 to –0.66) and increased Hb levels 0.59 g/L (95% CI, 0.38 to 0.81) compared with placebo. In patients with CKD, calcium-free phosphate binders were reported to be associated with a decreased risk of all-cause mortality compared with their calcium-based counterparts [23]. FC, a novel calcium-free phosphate binder, is approved by both US and Japan FDA since 2014. The present study revealed that FC is non-inferior to other active treatment including calcium- and non-calcium based phosphate binders in phosphate control. The results further provided evidence that FC reduced i-PTH and FGF 23, as well as increased Hb, serum ferritin, and TSAT. FC was associated with a less risk of constipation compared with sevelamer (OR, 0.12 [95% CI, 0.03–0.4], 3 of 116 participants for FC vs 21 of 113 participants for sevelamer). By contrast, FC was associated with a higher risk of diarrhea after 4 to 52 weeks of treatment (OR, 2.57 [95% CI, 1.29–5.15], 74 of 543 participants for FC vs 30 of 303 participants for placebo; and 21 of 161 participants for FC vs 1 of 158 participants for active treatments), whereas other serious adverse events remain uncertain.

Phosphate regulation is pivotal in CKD and dialysis patients. The serum phosphate reduction capacity of FC was similar to that of calcium carbonate, calcium citrate, and sevelamer carbonate [24]. The phosphorus-binding capacity of per gram of FC was estimated to be approximately 19.1 to 19.8 mg of phosphorus [25]. Hyperphosphatemia is a risk factor for cardiovascular risk and mortality with a direct stimulus of phosphorus resulting in disordered skeletal remodeling, heterotopic mineralization and vascular calcification [26, 27]. Phosphate binders are a treatment strategy to prevent or slow-down the progression of CKD-related mineral bone disease. Aluminum- and calcium-based phosphate binders have been used widely for a long time. The long-term use of aluminum hydroxide therapy can cause aluminum accumulation and toxicity. Calcium-based phosphate binders can result in hypercalcemia and metastatic calcification. The study results showed no substantial difference in serum calcium level between FC and active treatment. On the basis of data from three randomized controlled trials and 756 participants, we noted a trend towards a decrease in calcium change in ferric citrate group without statistical significance. The possible explanation for this in the analysis may be due to the active treatment included calcium containing compounds, sevelamer carbonate and sevelamer hydrochloride. New calcium- or aluminum-free new agents, sevelamer hydrochloride and lanthanum carbonate, have recently become available. These agents attenuate the progression of secondary hyperparathyroidism in patients with CKD [28]. Specific disadvantages of sevelamer hydrochloride and lanthanum carbonate include binding to other medications and increasing the medication cost.

A strong association has been reported between FGF-23 and a significantly elevated risk of all-cause mortality, cardiovascular events and initiation of chronic dialysis in patients with CKD [29–32]. Several studies have shown that phosphate binders reduces FGF-23 levels in patients with CKD and end-stage renal disease [33–35]. Furthermore, our meta-analysis revealed FC-induced reduction in FGF-23. Excess FGF-23 is a putative biomarker of cardiovascular disease [36]. It implies that therapeutic interventions targeting phosphate homeostasis by using FC can yield a useful intermediate marker of cardiovascular disease.

ESAs benefit patients with CKD free from frequent blood transfusion and associated complications. Observational studies have suggested that anemia in patients with CKD is independently associated with cardiovascular risk. However, the use of ESAs to increase Hb levels and subsequently reduce mortality risk remains uncertain [37–40]. In patients with CKD having iron deficiency anemia, iron supply aims to replenish iron stores and optimize Hb responses, thereby, improving their quality of life. The choice of oral versus intravenous iron supplementation depends on whether patients with CKD are receiving dialysis and the dialysis modality [41]. Observational studies have suggested that intravenous iron exacerbates oxidative stress, potentiates atherogenesis and cardiovascular toxicity, and increase the propensity for infections [42–44]. Adherence to oral iron is also greater with intravenous iron. FC was reported to be an effective oral phosphate binder that increases iron stores and reduces intravenous iron use and ESA dose [45]. No other oral irons were allowed during the study period in the RCTs analyzed in the present meta-analysis. Intravenous iron was administered in 5 trials but discontinued in case of high ferritin or TSAT levels. FC contains 210 mg/g elemental iron. The oral administration of iron is relative convenient in patients with CKD without dialysis and those receiving peritoneal dialysis.

Oral iron supplementation has side effects, such as epigastric discomfort, nausea, diarrhea, and constipation. Patients often pass black stool but it is not harmful. FC is administered with food, and hence result in fewer adverse gastrointestinal effects. FC was reported to be well tolerated in patients taking a dose of 1–8 g/day in a dose-dependent manner [46]. Consistent with previous studies, the present findings resulted as lower incidence of constipation after FC treatment than after sevelamer treatment. Furthermore, the citrate anion can be metabolized to bicarbonate to provide additional buffering for the daily ingested and generated acid load in patients with CKD.

The present meta-analysis has some strengths. We used rigorous methods and focused on the clinically important outcomes of CKD. Overall, our study revealed the potential of FC for in patients with CKD, particularly those with hyperphosphatemia and anemia. Calcium-free phosphate binders are costly; therefore, FC appears to be non-inferior in phosphate control compared to other non-calcium based phosphate binder and benefit in a concomitant correction of anemia in CKD patients. This systematic review does not replace the need for a well-conducted clinical trial. The present findings provide additional information relevant to the design of future studies. Treatment with FC appeared effectively alleviated anemia and reduced the incidence of constipation. Therefore, CKD patients with anemia and constipation might be preferred population in future trials.

Some study limitations should be acknowledged. First, a key limitation is the lack of data on outcomes of cardiovascular complications and mortality, which are particularly important in patients with CKD. Additional RCTs are required with a larger sample of patients with CKD who are and are not receiving dialysis. Longer follow-up and an assessment of the effects of FC on cardiovascular disease are necessary. Second, more trials are required to compare FC with other active treatments. Third, several outcomes demonstrated considerably high between-study heterogeneity. Part of reasons for this are due to that the concomitant use of ESA and intravenous iron could not be excluded. Moreover, the detailed dosages of intravenous iron were not available. Notably, this heterogeneity was quantitative, not qualitative.

In summary, the present systematic review of published RCTs suggested that FC reduces serum phosphate, i-PTH, and FGF-23 levels and improves anemia-related parameters, namely Hb, TSAT, and ferritin in patients with CKD stage 3–5D. Compared with placebo and active treatments, FC did not yield a higher incidence of adverse effects other than diarrhea. Additional RCTs are warranted to define the optimal population consideration cost-effectiveness and to determine FC intervention best offsets the risk of cardiovascular disease and mortality.

## MATERIALS AND METHODS

### Search Strategy

We used the Preferred Reporting Items for Systematic Reviews and Meta-Analyses statement, explanation and elaboration documents, and checklist to guide our methodology and reporting [47]. Studies were identified through a computerized search of the PubMed, EMBASE, and Cochrane databases. The following Medical Subject Headings were used: “dialysis,” “chronic kidney disease,” “end-stage renal disease,” “ferric citrate,” and “JTT-751.” Finally, unpublished trials were searched in the ClinicalTrials.gov registry (http://clinicaltrials.gov/). No language restrictions were applied in the selection of RCTs. The final search was performed in March 2017.

### Data Collection and Validity Assessment

Two reviewers (M.Y. Wu and C.H. Lin) independently extracted data on patient characteristics (age, sex, CKD stage with or without dialysis, and initial serum phosphate levels), inclusion and exclusion criteria, FC dosage, comparison groups, co-medications (particularly parenteral iron or ESAs), and follow-up duration. Considering the multiple measurements of the study end points [change in serum phosphate, calcium, i-PTH, FGF-23, Hb, iron, TSAT, and ferritin], the end point of interest in the present meta-analysis was defined as the change from the baseline to the last available measurement in the study. We included studies that enrolled adult patients (> 18 years) with CKD, defined as estimated glomerular filtration rate < 60 mL/min/1.73 m^2^, who received dialysis. Furthermore, studies had randomized patients to either the FC treatment group or control (placebo or active control) and reported at least one of the following outcomes: laboratory data and complications.

Analyses were performed including continuous analyses of differences in changes in serum phosphate, calcium, i-PTH, FGF-23, Hb, TSAT and ferritin, which yielded changes in parameters over time in the FC and comparison groups. Dichotomous analyses were performed, in which the proportion of patients with complications of constipation or diarrhea, occurring from the baseline to the last measurement, was the outcome of interest. The individually recorded decisions of the 2 reviewers were compared, and any disagreements were resolved by a third reviewer (Y.C. Chen). The corresponding author was contacted to request additional data when necessary.

The quality assessment of the included studies was assessed considering the following aspects: randomization adequacy, allocation concealment, investigators blinding, participant and outcome assessors, follow-up duration, drop-outs rate and whether intention-to-treat (ITT) analysis was conducted. Disagreement on eligibility and risk of bias were resolved disagreements by reviewer consensus.

### Statistical analysis

We used the following outcomes to evaluate the efficacy of FC in patients with CKD: changes in serum phosphate, calcium, i-PTH, FGF-23, Hb, TSAT, and ferritin from the baseline and to the occurrence of constipation or diarrhea.

We performed all statistical analyses using the statistical package Review Manager, Version 5.3 (Cochrane Collaboration, Oxford, England). We statistically analyzed the dichotomous outcomes by using ORs as the summary statistic. Continuous outcomes were analyzed using the weighted mean MD. When the mean or standard deviation values were unavailable, they were recalculated from other effect estimates and dispersion measures. We calculated the mean difference, which represents the combination of absolute differences between mean values in the two groups in a trial. Both types of summary statistics were reported with 95 % CIs. There were two models for meta-analysis. Fixed effect model assumed that treatment effects are common across studies; and random effects model assumed that treatment effects were heterogeneous across studies. A pooled estimate of the OR was computed using the DerSimonian and Laird random effects model, which provides a more appropriate estimate of the average treatment effect when trials are statistically heterogeneous, and typically yields wider CIs, thereby resulting in a more conservative statistical claim. For each meta-analysis, the Cochran Q statistic and I^2^ were calculated to assess the heterogeneity among the included studies. For Cochrane Q statistic of *p* < 0.1, the assumption of homogeneity was invalid. Furthermore, the causes of heterogeneity were explored in the context. We used the funnel plot to determine publications bias regarding the end points. Two-tailed *P* < 0.05 was considered statistically significant.
